# The Continuity of Scientific Discovery and Its Communication: The Example of Michael Faraday

**Published:** 2009-02-25

**Authors:** Alan G. Gross

**Affiliations:** 1 Professor of Communication Studies, 238 Ford Hall, University of Minnesota-Twin Cities, Minneapolis, MN 55455

## Abstract

This paper documents the cognitive strategies that led to Faraday’s first significant scientific discovery. For Faraday, discovery is essentially a matter seeing as, of substituting for the eye all possess the eye of analysis all scientists must develop. In the process of making his first significant discovery, Faraday learns to dismiss the magnetic attractions and repulsions he and others had observed; by means of systematic variations in his experimental set-up, he learns to see these motions as circular: it is the first indication that an electro-magnetic field exists. In communicating his discoveries, Faraday, of course, takes into consideration his various audiences’ varying needs and their differences in scientific competence; but whatever his audience, Faraday learns to convey what it feels like to do science, to shift from seeing to seeing as, from sight to insight.

## Preface

“There is,” says Richard Feynman, “a rhythm and a pattern between the phenomena of nature which is not apparent to the eye, but only to the eye of analysis.” In reflecting on scientific discovery, Feynman uses this metaphor to describe a process that is, in his view, essentially metaphorical, a constructive movement from seeing to seeing as, from sight to insight. This is also Faraday’s view of scientific discovery. In making his first significant electro-magnetic discovery, he learns to see apparent magnetic attraction and repulsion as, in reality, circular motion, the first hint of the existence of an electro-magnetic field. In literary experiments carried out in connection with this discovery, he makes another discovery, a discovery about scientific communication. He learns to recreate for his peers the feeling of discovery he has experienced. For Faraday, this strategy will apply across the board, whether his audience is professional or popular. While he must accommodate what he knows to the limited attention span and state of knowledge of the general audiences of his popular lectures, he is determined never to simplify to the point of omitting from his exposition what it is to make a scientific discovery. As we move from his Diary, tohis scientific papers, to his lectures to young people, we see a continuum in the way scientific discovery is conveyed. In all of these cases, Faraday leads us on a journey from seeing to seeing as, from sight to insight. 

## Faraday’s Discovery 

In 1821, at the age of thirty-one, a virtually unknown Michael Faraday published in the Quarterly Journal of Science his first significant discovery: the reciprocal circular motion of a magnet and an electric current, the first foray in the life-long enterprise of developing a field theory of magnetism and electricity. In a letter to Charles-Gaspard de la Rive, a Swiss professor of chemistry, dated 12 September, Faraday makes it clear that discovery for him is the coincidence of seeing and seeing as:

I find that all the usual attractions and repulsions of the Magnetic needle by the conjunctive wire are deceptions[,] the motions being not attractions &c or repulsions nor the result of any attractive or repulsive force but the results of a force in the wire which instead of bringing the pole of the needle nearer to or farther from the wire endeavours to make it move round it in a never ending circle and motion whilst the battery remains in action[.] I have succeeded not only in shewing the existence of this motion theoretically but experimentally and have been able to make the wire revolve round a magnetic pole or a magnetic pole round the wire at pleasure[.] The law of revolution and to which the other motions of the needle and wire are reducible is simple and beautiful [1, p. 222].

**Figure 1:**
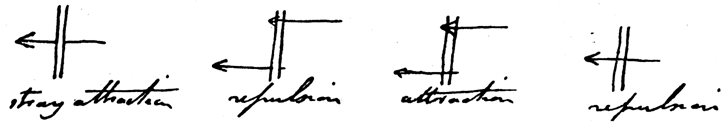
The needle is construed as moving toward and away from the wire.

Faraday’s exciting news was the result of a series of experiments initiated less than ten days earlier. On September 3, he made the following entry in his Diary concerning the position of a needle in the vicinity of a magnetized wire [2, pp. 49-50]: “Positions at first ascertained were as follows” (Figure 1 ).

**Figure 2:**
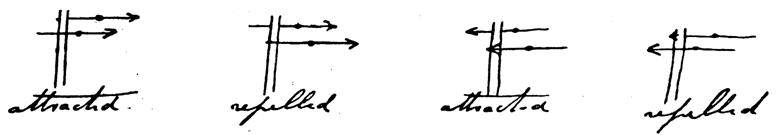
The wire is construed as having four positions per magnetic pole.

Faraday goes on: “ On examining these [positions] more minutely [I] found that each pole had [not 2 but] 4 positions, 2 of attraction, 2 of repulsion, thus” (Figure 2).

**Figure 3:**
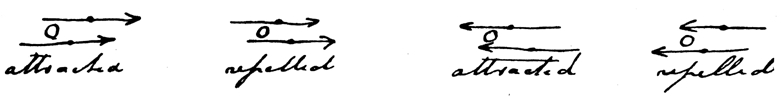
The wire in Figure 2 viewed from above.

Proceeding further, he says: “Or looking from above down on to sections of the wire [I saw]” (Figure 3).

**Figure 4:**
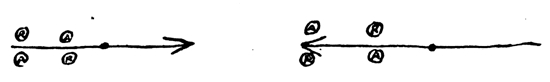
The needle is imagined as fixed, the wire as in motion. The positions of the wire can now be construed as forming points on a plane figure.

He goes on: “Or [alternately]” (Figure 4).

**Figure 5:**
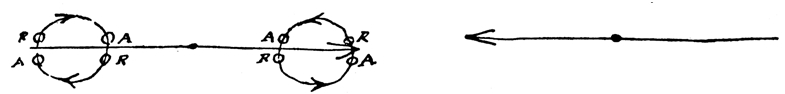
The wire is construed as circling the needle.

In a next step, Faraday imagines these points as positions on the circumference of a circle: “These [series’ of attractions and repulsions] indicate motions in circles round each [magnetic] pole, thus” (Figure 5).

Figure 5 constitutes a leap of faith, a move from sight to insight: what Faraday sees he sees as circular motion. The eye of analysis supervenes.

**Figure 6:**
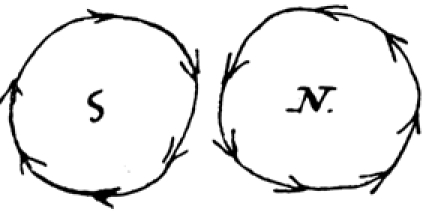
Circular motion actually observed.

When he fails actually to obtain the circular effect he has inferred, Faraday varies his apparatus: “[I] arranged a magnetic needle in a glass tube with mercury about it and by a cork, water, etc. supported a connecting wire so that the upper end should go into the silver cup and its mercury and the lower move in the channel of mercury round the pole of the needle. … In this way [I] got the revolution of the wire round the pole of the magnet. The direction was as follows, looking down from above” (Figure 6).

**Figure 7:**
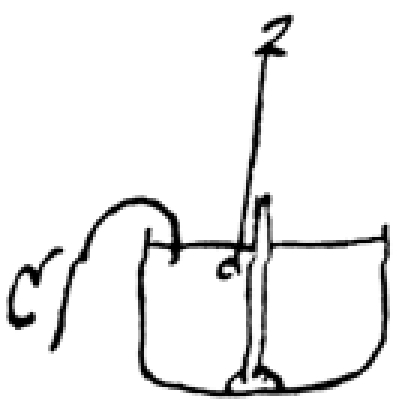
Apparatus designed to demonstrate the effect Faraday has intuited.

At the end of the day Faraday notes that the result is “very satisfactory,” but he decides to “make more sensible apparatus,” that is, one in which the effect he has observed is unequivocal. On the following day, Tuesday, September 4, Faraday does just that: “Apparatus for revolution of wire and magnet. A deep basin with a bit of wax at bottom and then filled with mercury, a Magnet stuck upright in wax so that pole just above the surface of mercury, then piece of wire floated by cork, at lower end dipping into mercury and above into silver cup as before, and confined by wire or capillary action from leaving M. Pole. Now Magnet round wire” (Figure 7).

**Figure 8:**
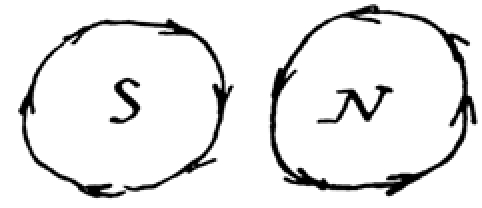
The effect that this apparatus demonstrates.

Faraday has constructed an apparatus that will be “more sensible,” that is, that will permit the unambiguous observation of the phenomenon of circular motion. In this figure, Z is the zinc connector; C is the copper connector [3; see also 4-7]. The apparatus achieves the desired confirmatory result (Figure 8).

The sketches in Figures 1 to 8 may best be understood in terms of David Gooding’s distinction between construal and interpretation. Construal is not an earlier stage of interpretation; rather

the term ‘construal’ is meant to draw attention to the dependence of construals on the context of action. … A construal cannot be grasped independently of the exploratory behaviour that produces it or the ostensive practices whereby an observer tries to convey it. Successfully communicated, it orders phenomena into an intelligible form that is less dependent upon operational or behavioural demonstration. Construals enable an ascent from the immediate and concrete world. [5, p. 87; his emphasis]

In this sequence of Diary entries,guided by words and empowered by visuals, Faraday moves from the representation of the apparent to the construal of the underlying reality that is his discovery. Consider the entry that precedes Figure 5, a ‘sentence’ that begin with words and ends with a picture. What is the purpose of the “thus”? When we look back at the words that precede it, we see a logical operator embedded in a clause; when we look forward to the depiction that follows, this same operator points to a conclusion that Faraday simultaneously asserts and sees—a visual inference. At this point, he devises a series of experiments to turn this visual inference into a theoretical interpretation. In a later paper [8, p. 303], he reveals the strategy behind this procedure: “guided by the idea of what ought to happen, supposing the cause now assigned were the true one, the following amongst many other experiments were made.” 

In this transformative process, the visual is always central: “it would be a mistake,” Gooding asserts, “to see [Faraday’s] drawings [of apparatus, its effects and the construal of those effects] simply as supplementing the text” [5 p. 123]. Rather, these drawings exemplify a principled division of labor between the verbal and the visual. Art historian William M. Ivins, Jr., puts his finger on the problem with sheer text in the representation and communication of meaning—the poverty of language as a medium for conveying accurate, as opposed to evocative, descriptions:

the moment that anyone seriously tries to describe an object carefully and accurately in words, his attempt takes the form of an interminably long and prolix rigmarole that few persons have either the patience or the intelligence to understand. A serious attempt to describe even a simple piece of machinery, such, let us say, as a kitchen can-opener with several moving parts, results in a morass of words that only a highly trained patent lawyer can cope with. [9, p. 57]

John Ziman’s insight into the role of the visual in scientific discovery is analogous:

A photograph, a tape-recording, an electronic device, can react to many causes simultaneously, and yet record the consequences as a complex pattern, accurately and reproducibly. It thus permits us to entertain theories and explanations whose workings and consequences cannot be represented by symbols placed in order on the page. [10, pp. 47-48]

Ryan Tweney’s comment on Faraday’s practice of discovery parallels Ziman’s claim:

Faraday enhanced the multiplicity of small events during the simultaneous moment of time. Here we can see the role that his preceding researches must have played in sensitizing him to the appearances he was seeking; it was a natural development to seek phenomena whose appearances could be deceptive. The task, which he succeeded in mastering, was to place the relatively slow acting perceptual system, the ‘eye,’ in a position to see what might be (and turned out in fact to be) fast acting events. Faraday succeeded … because, for him, the creation of a new order of natural law depended upon both cognitive and physical knowledge. [11, p. 164]

This reconstruction of Faraday’s practice is confirmed in a letter written toward the end of his life: “I was never able to make a fact my own without seeing it,” he says, “and the description of the best works altogether failed to convey to my mind, such knowledge of things as to allow myself to form a judgment on them.” He goes on to say that this is especially true of “new things [that is, discoveries]. If Grove or Wheatstone, or Gassiot, or any other told me a new fact & wanted my opinion, either of its value, or the cause, or the evidence it could give in any subject, I never could say any thing until I had seen the fact” [12, p. 975; his emphasis].

## Faraday Disseminates His Discovery

In his letter to de la Rive, as in his Diary, Faraday interpreted the circular motion he construed as a sign of a new fundamental force, clearly to be differentiated from the only forces previously recognized—Newtonian forces that can act only on bodies and can produce only motion in a straight line.In contrast, in his 1821 paper, Faraday needed not only to convey his discovery, but also to solicit the agreement of the relevant scientific community that his discovery was a discovery, that for them as for him seeing and seeing as coincided. He had to demonstrate that the reciprocal revolution of wire and magnet were natural expressions of a force fundamentally different from and irreducible to those discovered by Newton:

A scientific discovery is the public attribution of novelty to a claim regarded by the relevant scientific community as possible and as the consequence of following appropriate methods. It is the work of one or at most a small number of persons and takes place, ideally, in an instant of time. These criteria are contingent; nevertheless, once in place, they behave just like conditions that are individually necessary and jointly sufficient for the attribution of scientific discovery. [13, p. 168; emphasis omitted]

In his 1821 paper, however, Faraday has not brought to perfection the literary techniques that will convey unequivocally to his peers the phenomenological law he has so perspicuously intuited. True, he has made significant progress along this path: in the interest of clarity, he has deliberately simplified the circuitous route by which he actually achieved his discovery “in such a manner as to give the most concise view of the whole.” This is because in his literary endeavors he considers himself “at liberty to rearrange [the order of events] in a manner calculated to convey most readily what appears to me to be a correct view of the nature of the phenomena” [see 5, end-papers; 14, pp. 2, 25]. Nevertheless, in this early paper, his complex narrative of trial and error overburdens the reader who must, without the assistance of diagrams, visualize, sequentially, the initial apparatus, its modification, and its final state (see Appendix 1). 

Gooding does not see this omission as a defect; indeed, he says of this absence that “the literary account places phenomena in an objective relationship to theories just as the material embodiment of the skills places phenomena in an objective relationship to human experience. This is why this first paper illustrates the circles of rotation independently of an image of the apparatus” [5, p. 177]. But Faraday himself sees this omission as an error, one he corrects in the second of two addenda published subsequent to his paper. 

**Figure 9:**
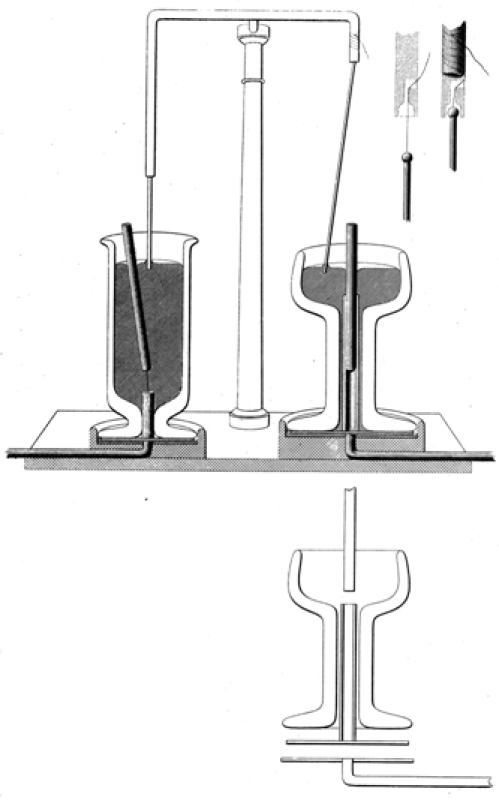
X-ray views make the inner workings of the apparatus transparent.

In his first addendum (Appendix 2), he tries once again to convey the essence of his discovery. But while he describes, he still does not depict the apparatus that will demonstrate his effect. Moreover, his method of description still does not serve his purpose; he emphasizes structure over operation, treating his apparatus as if it were a building, rather a window transparent to nature’s fundamental forces. In a second addendum (Appendix 3), he corrects these initial missteps. After admitting that the account in the first was “imperfect,” he accompanies a depiction of his apparatus with a description that focuses precisely on its operation: the order of his prose foregrounds its active components, creating an overarching narrative at whose inception there is the electrical current and at whose climax the desired revolution takes place, a tale that moves transparently from cause to effect. Moreover, in the second addendum, not only is the apparatus depicted, but so are its components whose structure might otherwise be hidden from view. These described and depicted details are especially important: they reveal that nowhere in the apparatus’ construction is there any barrier to the unmediated realization of the natural effects that are the essence of his discovery. Faraday’s depictions of his apparatus are, of course, realistic representations. In the semiotic terminology of the philosopher Charles Sanders Peirce, these depictions are iconic. But they are iconic only in the interest of indexicality: they allow us to see through his apparatus so that we can see the causal structure of the world as it really is (Figure 9).

**Figure 10:**
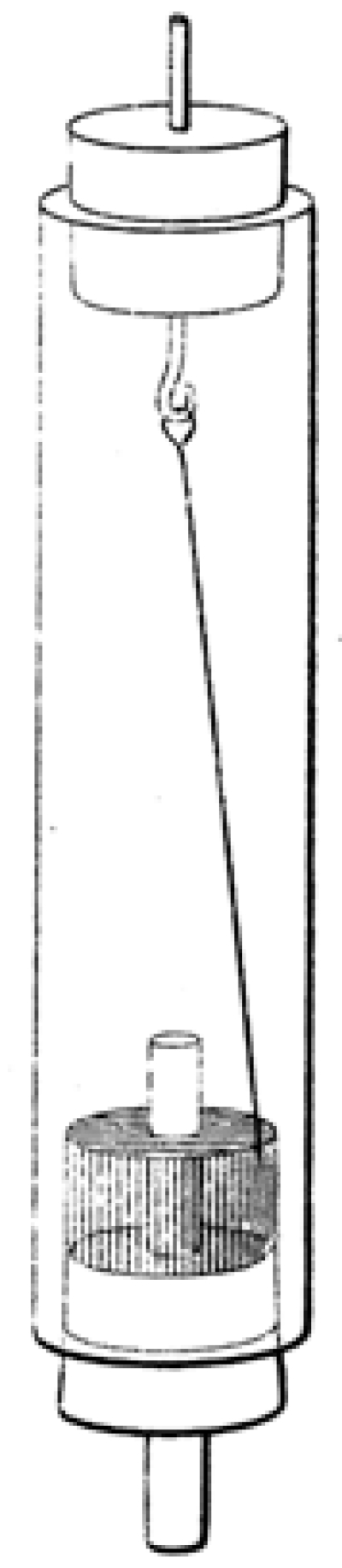
An apparatus that guarantees the replication of the crucial experiment.

No depiction, no matter how accurate, and no description, no matter how complete, can guarantee replicability, can guarantee, that is, that seeing and seeing aswill coincide for both the discoverer and his chosen audience. In this matter, seemingly, Faraday was at the mercy of the skills of others. Of course, at the Royal Institution he could demonstrate his effect. Nevertheless, he could not be sure that his fellow Englishmen far from London, or that his Continental colleagues, would accurately duplicate his crucial experiment, a task made especially difficult because of the delicacy of the manipulations involved. Nor was it practicable for him to turn from working scientist to peripatetic demonstrator. Faraday meets this challenge with his usual ingenuity. He does not travel; his experiment does. His original apparatus is too large—and too expensive—to disseminate to his fellow scientists who could not be present at his lectures. Consequently, he creates a “pocket” version of this apparatus, a portable means of persuasion, one he depicts and describes thoroughly in his second addendum. Bruno Latour explains this process: “the history of technoscience is in large part the history of all the little inventions made along the networks to accelerate the mobility of traces, or to enhance their faithfulness, combination, and cohesion, so as to make action at a distance possible” [15, p. 254] (Figure 10).

As Faraday’s apparatus moves by Royal Mail along the communicative network that he creates, it forms filiations of believers in the reality of his effect and its cause. For example, the French physicist and mathematician, Jean Nicolas Pierre Hachette, writes to Faraday that he “received the two copies of your paper on electro-magnetism as well as the little instrument.” Faraday sends the same apparatus to de la Rive with instructions for its use. Subsequent to this, Hachette and Ampère “repeat[] your lovely experiment.” In December, Ampère gives an account of and demonstrates this experiment at the Royal Academy of Sciences [1, pp. 234-43]. 

I do not know whether Faraday was aware of Newton’s problems in disseminating the results of his early optical discoveries. The parallel is, nonetheless, instructive. It was not until the Opticks in 1704 that, through words and pictures, Newton communicated a recipe for revealing the color homogeneity he had demonstrated to his own satisfaction thirty-two years earlier; it was not until 1714, in response to a Continental challenge, that Desaguliers, under Newton’s direction, demonstrated color homogeneity in public. In Simon Schaffer’s words:

Desaguliers tailored his experiments for effective witnessing. Spectators were each given a hand-held prism through which to view the spectrum cast on a final screen. …. After a dry run at his house in Westminster, Desaguliers showed them to the [Royal] Society. In early 1715, they were displayed to visiting natural philosophers from Holland, Italy, and France. [16, pp. 95-96; see also 17 for a critique of Schaffer that does not, however, contest this account]

Finally, after nearly a half century, seeing and seeing as coincided for both Newton and his audience. 

The replication of scientific discoveries remains a formidable challenge, even today; it was even more difficult in former times when apparatus was so often made by the experimenter himself or made to order according to his instructions. Faraday recognized this problem and solved it in a manner Newton might have envied. As a result of his traveling apparatus, scientists in England and on the Continent viewed exactly what he viewed exactly in the way he viewed it. Where there might have been interpretive dissensus, there was now interpretive consensus that pointed to the existence of a hitherto undiscovered force in the universe (Ampère, however, was not convinced; see 18, p. 96).

In transforming the verbal and the visual resources at his disposal into the narrative, descriptive, and argumentative structures that constitute the 1821 paper and its appendices, Faraday has invented a literary technology adequate to communicating his scientific to his peers. Recognizing the inherent limitations of all literary technologies, however, he has also invented apparatus that turns his discovery into a discovery all can witness and acknowledge. By means of literary techniques, Faraday creates a straightforward path from experiment to discovery; by means of apparatus, he permits his peers to experience what he experienced at the crucial moment of insight. 

## Faraday Popularizes His Discovery

The literary strategy Faraday developed in communicating his first significant discovery to his peers is also the animating force behind his numerous popular lectures. In a series of letters to Benjamin Abbott in June, 1813, he comments on the role of visuals and apparatus in public lectures:

Apparatus therefore is an essential part of every lecture in which it can be introduced but to apparatus should be added at every convenient opportunity illustrations that may not perhaps deserve the name of apparatus and of experiments and yet may be introduced with considerable force and effect in proper places. Diagrams & Tables too are necessary or at least in an eminent degree to the illustration and perfection of a Lecture. [1, p. 59]

Still, Faraday’s public lectures would be seriously incomplete if they merely demonstrated effects. These effects must also be accompanied by theory: to discover is also to explain. Faraday is a scientist, not a mountebank: “apt experiments,” he says, “ought to be explained by a satisfactory theory or otherwise we merely patch an old coat with new cloth and the whole (hole) becomes worse” [1, p. 65].

**Figure 11:**
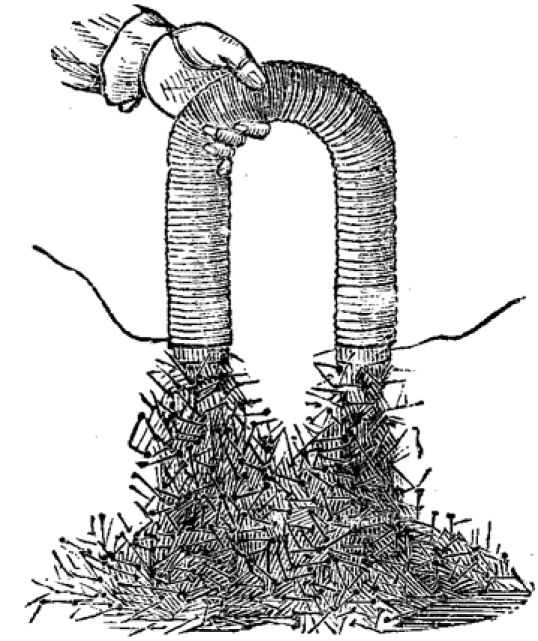
Faraday’s demonstration makes visible the transformation of chemical into electrical energy and then into magnetism. Note the two solitary wires connecting the horseshoe to a battery.

These convictions are fully embodied in Faraday’s lectures to young people. In a lecture on electromagnetism,for example, he makes his explanatory purpose abundantly clear: “we have not merely to see how it is that one power affects another—how the force of heat affects chemical affinity and so forth—but we must try and comprehend what relation they bear to each other, and how these powers may be changed into other” [19,p. 144]. This statement is followed by a demonstration showing the transfer of the chemical energy of a battery into electrical energy that magnetizes an iron horseshoe. Here, as in his Diary and in his 1821 article, the iconic is interpreted as an index of the presence of fundamental forces (Fig. 11).

Faraday concludes his lecture by extracting a scientific moral from his demonstration, an effortless shift from show to prove:

What, then, can surpass these evidences of the change of chemical force into electricity, and electricity into magnetism? I might show you many other experiments whereby I could obtain electricity and chemical action, heat and light, from a magnet, but what more need I show you to prove the universal correlation of physical forces of matter, and their mutual conversion into another? [199, p. 166; emphasis mine]

In such lectures, because Faraday is not speaking to scientists, he does not trouble his audience with “minutiae” of experimental method or with a train of reasoning as complex as those exhibited in his scientific papers [20, p. 71]. Nevertheless in his popular lectures he is always concerned to represent the reasoning behind scientific discovery: “The reason why I make the experiment in this manner is solely that I may cause the steps of our demonstration to be so simple that you can never for a moment lose the train of reasoning, if only you pay attention” [20, p. 69]. Of this at least one member of his audience, Lady Pollock, was well aware: “He never suffered an experiment to lead him away from his theme. Every touch of his hand was a true illustration of his argument” [21, pp. xxii-xxiii].

Faraday is firm in his conviction that communicating to a general audience is an endeavor just as responsible as communicating to their professional counterparts or, for that matter, communing with his future self in his Diary: all these processes center around the reciprocity between showing and proving. Toward the end of an 1831 paper on electromagnetic induction, for example, after stating the law he has discovered, Faraday draws a diagram illustrating its operation. At this point, he says that this law may also be explained “in a popular way” by means of another diagram he provides, in which the magnetic lines of force are “cut” by a silver (non-magnetic) pen-knife. Finally, he asserts, “a little model is easily constructed, by using a cylinder of wood for a magnet, a flat piece for the blade, and a piece of thread connecting one end of the cylinder with the other, and passing through a hole in the blade, for the magnetic curves; this readily gives the result of any possible direction” [14, paragraph 116]. Howard Fisher rightly emphasizes the continuity among these representations: he affirms that this “suggestion should be seen as a small example of Faraday’s evolutionary representations of phenomena—passing in this case from a rule or “law” …to a diagram … to the knife blade image, to an actual, tangible device. Notice the increasing visible presence of these successive representations” [23, p. 68n; his emphasis]. 

## Conclusion

For Faraday, discovery and dissemination form a continuum, a variety of ways of instantiating and recording the reasoning behind his science. It is by means of these processes that the causal structure of the world is revealed to varying audiences, a revelation always construed as the coincidence of seeing and seeing as. For Faraday, deference to the needs and capacities of his audience was no mere façon de parler. Still, all of his communicative practices, while they are fine-tuned to an audience’s needs and capacities, are analogous in approach and method. In each genre, Faraday takes his audience on a metaphoric journey as he moves from seeing to seeing as, from sight to insight. Barbara McLintock asserted that she had “a feeling for the organism”; analogously, it may be said that Faraday had a feeling for the electrical field, a feeling that he strove to convey to his varying audiences throughout a long career.

## Appendices

### Appendix 1: Description of the Evolution of Equipment

The revolution of the wire and the pole round each other being the first important thing required to prove the nature of the force mutually exerted by them, various means were tried to succeed in producing it [23] . The difficulty consisted in making a suspension of part of the wire sufficiently delicate for the motion, and yet affording sufficient mass of matter for contact. This was overcome in the following manner: A piece of brass wire had a small button of silver soldered onto its end, a little cup was hollowed in the silver, and the metal being amalgamated, it would then retain a drop of mercury in it, though placed upside down for an upper centre of motion; for a lower centre, a similar cup was made of copper, into which a little mercury was put; this was placed in a jar of water under the former centre. A piece of copper wire was then bent into the form of a crank, its ends amalgamated, and the distances being arranged, they were placed in the cups. To prevent too much friction from the weight of the wire on the lower cup, it had been passed through a cork duly adjusted in size, and that being pushed down on the wire till immersed in the water, the friction became very little, and the wire very mobile, yet with good contacts. The plates being then connected with the two cups, the apparatus was completed. In this state, a magnetic pole being brought to the centre of motion of the crank, the wire immediately made an effort to revolve until it struck the magnet, and that being rapidly brought round to the other side, the wire again made a revolution, giving evidence that it would have gone round continually but for the extension of the magnet on the outside. To do away with this impediment, the wire and lower metal cup were removed, and a deep basin of mercury placed beneath; at the bottom of this was a piece of wax, and a small round bar magnet was stuck upright in it, so that one pole was about half or three-fourths of an inch above the surface of the mercury, and directly under the silver cup. A straight piece of copper wire long enough to reach from the cup, and dip about a half inch into the mercury, had its ends amalgamated, and a small round piece of cork fixed on to one of them to make it more buoyant; this being dipped in the mercury close beside the magnet, and the other end placed under the little cup, the wire remained upright, for the adhesion of the cork to the magnet was sufficient for that purpose, and yet at its lower end had freedom, of motion round the pole. The connection being now made from the plates to the upper cup, the wire immediately began to revolve round the pole of the magnet, and continued to do so as long as the connection was continued.

### Appendix 2: Electro-magnetic Rotation Apparatus [23]

Since the paper in the preceding pages has been printed, I have had an apparatus made by Mr. Newman, of Lisle Street, for the revolutions of the wire round the pole, and a pole around the wire. When Hare’s calorimot[o]r was connected with it, the wire moved so rapidly around the pole that the eye could scarcely follow the motion, and a single galvanic trough, containing ten pairs of plates, of Dr. Wollaston’s construction, had power enough to move the wire and the pole with considerable rapidity. It consists of a stand, about 3 inches by 6, from one end of which a brass pillar rises about six inches high, and is then continued horizontally by a copper rod over the stand; at the other end of the stand a copper plate is fixed with a wire for communication, brought out to one side; in the middle is a similar plate and a wire; these are both fixed. A small shallow glass cup, supported on a hollow foot of glass has a plate of metal cemented to the bottom, so as to close the aperture and form a connection with the plate on the stand; the hollow foot is a socket, into which a small cylindrical bar magnet can be placed, so that the upper pole shall be a little above the edge of the glass; mercury is then poured in until the glass is nearly full; a rod of metal descends from the horizontal arm perpendicularly over this cup; a little cavity is hollowed at the end, and amalgamated, and a piece of stiff copper wire is also amalgamated, and placed in it, as described in the paper, except that it is attached by a piece of thread in the manner of a ligament, passing from the end of the wire to the inner surface of the cup; the lower end of the wire is amalgamated, and furnished with a small roller, which [the wire] dips so as to be under the surface of the mercury in the cup beneath it.

The other plate on the stand has also its cup, which is nearly cylindrical, a metal pin passes through the bottom of it, to connect by contact with the plate below, and to the inner end of the pin a small round bar magnet is attached at one pole by thread, so as to allow the other to be above the surface of the mercury when the cup is filled, and have freedom of motion there; a thick wire descends from the rod above perpendicularly so as to dip a little way into the mercury of the cup; it forms the connecting wire, and the pole can move in any direction round it. When the connections are made with the pillar, and either of the wires from the stand plates, the revolution of the wire, or the pole above, takes place; or if the wires be connected with the two coming from the plates, the motion takes place in both cups at once, and in accordance with the law stated in the paper. This apparatus may be much reduced in size, and made very much more delicate and sensible.

### Appendix 3: Description of an Electro-magnetical Apparatus for the Exhibition of Rotary Motion [23]

The account given in the Miscellanea of the last Journal … of the apparatus invented in illustration of the paper in the body of that number, being short and imperfect; a plate is given in the present number, presenting a section of that apparatus, and a view of the smaller apparatus [Figure 10], illustrative of the motions of the wire and the pole around each other. The larger apparatus is delineated [Figure 9] on a scale of one half. It consists of two glass vessels, placed side by side with their appendages. In that on the left side of the plate the magnetic motion round the connecting wire of the voltaic battery is produced. That a current of voltaic electricity may be established through this cup, a hole is drilled at the bottom, and into this a copper pin is ground tight, which projects upward a little way into the cup, and below is riveted to a small round plate of copper, forming a part of the foot of the vessel. A similar plate of copper is fixed to the turned wooden base on which the cup is intended to stand, and a piece of strong copper wire, which is attached to it beneath, after proceeding downwards a little way, turns horizontally to the left hand, and forms one of the connections. The surfaces of these two plates intended to come together, are tinned and amalgamated, that they may remain longer clean and bright, and afford better contact. A small cylindrical and powerful magnet has one of its poles fastened to a piece of thread, which, at the other end, is attached to the copper pin at the bottom of the cup; and the height of the magnet and the length of the thread are so adjusted that when the cup is nearly filled with clean mercury the free pole shall float almost upright on its surface.

A small brass pillar rises from the stand behind the glass vessels; an arm comes forward from the top of it, supporting at its extremity a cross wire, which at the place on the left hand, where it is perpendicularly over the cup just described, bends downwards, and is continued till it just dips into the centre of the mercurial surface. The wire is diminished in size for a short distance above the surface of the mercury, and its lower extremity amalgamated, for the purpose of ensuring good contact; and so also is the copper pin at the bottom of the cup. When the poles of a voltaic apparatus are connected with the brass pillar, and with the lateral copper wire, the upper pole of the magnet immediately rotates round the wire which dips into the mercury; and in one direction or the other, according as the connections are made.

The other vessel is of the form delineated in the plate. The stem is hollow and tubular; but, instead of being filled by a plug, as is the aperture in the first vessel, a small copper socket is placed in it, and retained there by being fastened to a circular plate below, which is cemented to the glass foot, so that no mercury shall pass out by it. This plate is tinned and amalgamated on its lower surface, and stands on another plate and wire, just as in the former instance. A small circular bar magnet is placed in the socket, at any convenient height, and then the mercury poured in till it rises so high that nothing but the projecting pole of the magnet is left above its surface at the centre. The forms and relative positions of the magnet, socket, plate, &c., are seen in [Figure 9]

The cross wire supported by the brass pillar is also prolonged on the right hand, until over the centre of the vessel just described; it then turns downwards, and descends about half an inch; it has its lower extremity hollowed out into a cup, the inner surface of which is well amalgamated. A smaller piece of copper wire has a spherical head fixed on to it, of such a size that it may play in the cup in the manner of a ball and socket-joint, and being well amalgamated, it, when in the cup, retains sufficient fluid mercury by capillary attraction to form an excellent contact with freedom of motion. The ball is prevented from falling out of the socket by a piece of fine thread, which, being fastened to it at the top, passes through a small hole at the summit of the cup, and is made fast on the outside of the thick wire. This is more minutely explained by [the upper left diagram in Figure 9]. The small wire is of such length that it may dip a little way into the mercury, and its lower end is amalgamated. When the connections are so made with the pillar and the right-hand wire, that the current of electricity shall pass through this moveable wire, it immediately revolves around the pole of the magnet, in a direction dependant on the pole used, and the manner in which the connections are made.

Figure 10 is the delineation of a small apparatus, the wire in which moves rapidly, with very little voltaic power. It consists of a piece of glass tube, the bottom part of which is closed by a cork, through which a small piece of soft iron wire passes, so as to project above and below the cork. A little mercury is then poured in, to form a channel between the iron wire and the glass tube. The upper orifice is also closed by a cork, through which a piece of platinum wire passes which is terminated within by a loop; another piece of wire hangs from this by a loop, and its lower end, which dips a very little way into the mercury, being amalgamated, it is preserved from adhering either to the iron wire or the glass. When a very minute voltaic combination is connected with the upper and lower ends of this apparatus, and the pole of the magnet is placed in contact with the external end of the iron wire, the moveable wire within rapidly rotates round the magnet thus formed at the moment; and by changing either the connection, or the pole of the magnet in contact with the iron, the direction of the motion itself is changed.

The small apparatus in the plate is not drawn to any scale. It has been made so small as to produce rapid revolutions, by the action of two plates of zinc and copper, containing not more than a square inch of surface each.

In place of the ball and socket-joint [of the upper left diagram in Figure 9] loops may be used; or the fixed wire may terminate in a small cup containing mercury, with its aperture upwards, and the moveable wire may be bent into the form of a hook, of which the extremity should be sharpened, and rest in the mercury at the bottom of the cup.
